# A phase I pharmacokinetic and pharmacodynamic study of BGC9331 and carboplatin in relapsed gynaecological malignancies

**DOI:** 10.1038/sj.bjc.6602811

**Published:** 2005-10-11

**Authors:** T Benepal, A Jackman, L Pyle, S Bate, A Hardcastle, W Aherne, F Mitchell, L Simmons, R Ruddle, F Raynaud, M Gore

**Affiliations:** 1 Department of Medicine, The Royal Marsden Hospital, 203 Fulham Road, London SW3 6JJ, UK; 2 Section of Medicine, The Institute of Cancer Research, 15 Cotswold Road, Belmont, Surrey SM2 5NG, UK; 3 Cancer Research UK Centre for Cancer Therapeutics at The Institute of Cancer Research, 15 Cotswold Road, Belmont, Surrey SM2 5NG, UK

## Abstract

BGC9331 is a rationally designed, specific nonpolyglutamatable thymidylate synthase (TS) inhibitor that is active in gynaecological malignancies. In the light of the sensitivity of human ovarian tumour cell lines to BGC9331 and non-cross resistance to platinum drugs, we studied the combination BGC9331/carboplatin (BCA) in a phase I (PI) pharmacokinetic (PK) and pharmacodynamic (PD) study in platinum pretreated gynaecological malignancies. Patients were ⩾18 years or over, with a histologically confirmed gynaecological malignancy, radiological evidence of relapse, and a platinum treatment free interval of at least 6 months. Up to three prior lines of chemotherapy were permitted. Carboplatin (AUC5) and BGC9331 were administered on day 1, and BGC9331 was also given on day 8 of a 21-day cycle. In total, 14 patients were enrolled, and treated with BGC9331 at four dose levels, 40, 65, 85 and 100 mg m^−2^. The principal grade 3 and 4 haematological toxicity was neutropaenia. The principal nonhaematological toxicities were lethargy and nausea. Dose-limiting toxicities were seen in two patients at 100 mg m^−2^ BGC9331 (grade 4 neutropaenia >7 days, and grade 4 fatigue >7 days). Plasma BGC9331 was measured by an ELISA that was adapted for use in humans. Carboplatin was assayed by flameless atomic absorption spectrometry. There was no PK interaction between the two drugs. Plasma deoxyuridine was elevated indicating TS inhibition to at least day 12. Antitumour activity was observed in four out of 14 (28%) of patients. In conclusion, the combination of BGC9331 and carboplatin is well tolerated with no significant PK interaction between the two drugs. There is evidence of TS inhibition with the combination. We have demonstrated antitumour activity in platinum pretreated gynaecological malignancy. Further exploration of this combination in this disease is warranted.

## BGC9331 IN OVARIAN CANCER

Ovarian cancer remains the leading cause of death from gynaecological malignancy in the United Kingdom, affecting approximately 6000 women per year (CRC, 2001). The overall survival for this disease has changed little over the last few years, with the majority of women still relapsing and dying. Significant changes in the treatment of ovarian cancer have occurred over the last 30 years with the introduction into clinical practice of platinum-based regimes, and more recently in combination with taxanes. Platinum–taxane combinations are now regarded as standard first-line treatment of this disease. Current clinical trials are exploring combinations of platinum and taxanes with new agents as well as investigating sequential regimens.

BGC9331 is a rationally designed, quinazoline-based specific inhibitor of thymidylate synthase (TS) that was developed at the Institute of Cancer Research (ICR), Sutton, Surrey, UK in collaboration with AstraZeneca (current drug development under control of BTG International). It has been assessed in phase I/II studies in a number of tumour types including ovarian cancer (Plummer *et al*, 2003; Rader *et al*, 2003). Low clinical activity was previously demonstrated when targeting TS in ovarian cancer with the polyglutamatable TS inhibitor raltitrexed (RTX) demonstrating 7 and 11% response rates (Gore *et al*, 1995; Muggia *et al*, 1998), and 5-FU a 14% response rate (Look *et al*, 1995) in relapsed disease. However, there are a number of reasons why the addition of carboplatin to the nonpolyglutamatable TS inhibitor BGC9331 warrants investigation in this disease.

Firstly, CB3717, the first specific folate-based TS inhibitor to be developed clinically, RTX (Kelland *et al*, 1995) and BGC9331 (Jackman *et al*, 2002) have demonstrated non crossresistance to platinum-based agents in human ovarian tumour cell lines. Secondly, preclinical studies suggested that folyl-polyglutamate synthase (FPGS) expression, the enzyme required for polyglutamation of RTX but not BGC9331, may be lower in human ovarian tumour cell lines compared with human colorectal cancer cell lines and BGC9331 overcomes the associated resistance to RTX in these cells (Jackman *et al*, 2002). Thirdly, CB3717 demonstrated activity in platinum-resistant ovarian cancer (Calvert *et al*, 1986; Clarke *et al*, 1993) in phase I and phase II trials. This drug is a relatively poor substrate for FPGS compared to RTX (Jackman *et al*, 1993). Fourthly, *α*-folate receptor (*α*-FR) mediated uptake may contribute to the activity of some antifolates, particularly BGC9331 (Theti and Jackman, 2004). The *α*-FR is overexpressed in up to 90% of epithelial ovarian cancers (Toffoli *et al*, 1997). Finally, BGC9331 has demonstrated encouraging phase I and II activity in relapsed ovarian cancer (Plummer *et al*, 2003; Rader *et al*, 2003).

BGC9331 has been combined with cisplatin in a heavily pretreated PI population of patients with a variety of malignancies (Bilenker *et al*, 2004). Carboplatin, however, has little or no ototoxicity, neurotoxicity, nephrotoxicity and a reduced incidence of nausea and vomiting compared with cisplatin (Calvert *et al*, 1985). The lack of nephrotoxicity precludes the need for prolonged admission, allowing administration over 1 h as opposed to 8–12 h for cisplatin. Other phase I combination studies with BGC9331 have been undertaken with agents with activity in ovarian cancer (Louvet *et al*, 2002; Wood *et al*, 2002; Benson *et al*, 2003). This study is the first to combine a TS inhibitor with a platinum-based drug in ovarian cancer.

The primary aim of this study was to determine the maximum tolerated dose (MTD) of a combination of BGC9331 and carboplatin in platinum-sensitive gynaecological malignancies. Rational clinical development of any new agents should include pharmacodynamic (PD) end points, and therefore secondary aims in this study were the pharmacokinetics (PKs) of the combination, and the assessment of TS inhibition by measuring plasma deoxyuridine (dUrd), a nucleotide that increases following TS inhibition (Ford *et al*, 2002).

## PATIENTS AND METHODS

### Definitions

Dose-limiting hematological toxicities were defined as nadir absolute neutrophil count (ANC) <0.5 × 10^9^ l^−1^ for ⩾7 days, thrombocytopenia <25 × 10^9^ l^−1^, or an ANC <0.5 × 10^9^ l^−1^ associated with fever. Dose-limiting non-haematological toxicities were defined as any grade 3 or 4 toxicity not ameliorated by symptomatic measures or treatment delay ⩾2 weeks.

The MTD was defined as the dose at which two or more patients experienced dose-limiting toxicities.

### Patient recruitment and eligibility

Patients were recruited from the Royal Marsden Hospital (RMH) Gynaecological Oncology Unit between September 2000 and June 2002.

Women who were ⩾18 years, with a performance status (PS) of 0–2 and a life expectancy of >3 months were entered into the study. Patients required histologically or cytologically confirmed adenocarcinoma of the ovary, peritoneum or fallopian tube, mixed mullerian tumour, or other gynaecological malignancy for which the use of carboplatin or TS inhibitor in relapsed disease would be considered reasonable. A platinum therapy free interval of ⩾6 months was required prior to initiation of trial therapy. Patients were excluded for the following reasons: progression on or within 6 months of the end of platinum treatment, no change in disease status at the end of platinum treatment, or response after three cycles of platinum treatment but with no further response after six cycles.

Patients were allowed up to three prior lines of chemotherapy, but those who had received previous or concomitant radiotherapy were excluded. No previous exposure to TS inhibitors or previous hormonal or chemotherapy within 4 weeks (no exposure to nitrosureas or mitomycin C within 6 weeks) of initiation of treatment was permitted. Minimal haematological parameters were as follows: neutrophils ⩾1.5 × 10^9^ l^−1^, haemaglobin ⩾9g dl^−1^, platelets ⩾100 × 10^9^ l^−1^. Adequate liver function as demonstrated by serum bilirubin ⩽1.25 × upper limit of reference range (ULRR), and/or alanine aminotransferase (ALT) or aspartate aminotransferase (AST) ⩽5 × ULRR in the presence of liver metastases, or ⩽2.5 × ULRR in the absence of demonstrable liver metastases. Albumin was required to be ⩾lower limit of normal. Minimal renal function was a creatinine clearance (GFR) of ⩾60 ml min^−1^, as calculated by the EDTA/Cockcroft-Gault equation. Written informed consent to participate in the trial was obtained prior to treatment, according to guidelines set out by the institutions ethics committee, which approved the study.

### Trial design

**Figure 1:**
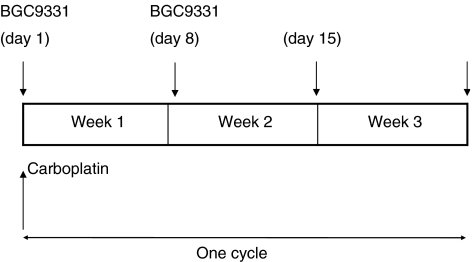
Dose administration schedule of BGC9331 and carboplatin.

This was a phase I dose escalation study of BGC9331 in gynaecological malignancies with a fixed dose of carboplatin in patients with greater than 6 months platinum free treatment period. Three patients were planned at each dose level and the MTD was defined as the dose at which two or more patients experienced dose-limiting toxicities. The dose level below MTD was to be expanded to six patients. There were six dose levels in the trial. The carboplatin was administered at a fixed dose of AUC5, as determined by the formula: AUC=(glomerular filtration rate (GFR)+25) × 5 (Calvert *et al*, 1989) at all dose levels. The GFR was determined in all patients by ^51^CrEDTA (Chantler *et al*, 1969). The planned starting BGC9331 dose was 40 mg m^−2^, then escalated to 65, 85, 100 and finally 130 mg m^−2^, the recommended single-agent phase II dose (Plummer *et al*, 2003) (Table 1). As a precaution, a reduced BGC9331 dose level was included in the event of severe toxicities being observed at early dose levels (30 mg m^−2^). The carboplatin was administered at AUC5 on day 1 of a 21-day cycle, with BGC9331 administered on days 1 and 8 of a 21-day cycle (Figure 1).

### Assessment methods

#### Pretreatment evaluation

Patients were assessed prior to initiation of therapy by physical examination and full blood count (FBC), urea and electrolytes (U&E) and liver function tests (LFT), computerised axial tomography (CT), serum tumour marker measurement (CA-125), and electrocardiograph (ECG).

#### Toxicity

Following initiation of treatment an FBC was performed weekly, with U&E and LFT and full clinical examination performed prior to day 1 of each new cycle of chemotherapy, or when clinically indicated. Toxicity was assessed according to NCI-EORTC toxicity criteria, prior to each new cycle of treatment or when clinically indicated.

#### Response

Response was assessed radiologically following every two cycles of chemotherapy according to WHO criteria (Miller *et al*, 1981) or when clinically indicated. CA-125 was measured prior to each new cycle of chemotherapy.

#### PK and PD measurements

Blood was taken for carboplatin assessment pretreatment on day 1 and then 0.5, 1, 1.5, 2, 4, 6, 9, 12, 24, 48, 72, 96 and 168 h after treatment. Blood was taken for BGC9331 pretreatment and then 0.5, 1, 1.5, 2, 3, 4, 6, 9, 12, 24, 48, 72, 96, 168, 168.5, 169, 170, 171, 172, 174, 176, 180, 192, 216, 240 and 264 h following treatment. Blood was also taken for dUrd assessment at 4, 9, 12, 24, 48, 96, 168, 192 and 264 h.

### Measurement of BGC9331 by ELISA

#### Adaptation of the ELISA assay for analysis of human plasma

The BGC9331 in this study was assayed by ELISA that has been previously described for use with animal samples (Aherne *et al*, 2001). The assay has never previously been used to measure drug levels in humans. Prior to each assay the plasma samples were centrifuged at 3000 g for 5 min × 2, to sediment precipitants that would block pipette tips. Serial dilutions were 10–20 times greater than required for measurement of drug levels in *in vitro* cultured cells or *in vivo* mouse samples, and were selected so that at least one dilution per sample would fall within the standard curve.

All dilutions required for the ELISA were performed by Multiprobe II automated liquid handling equipment (Perkin-Elmer, Cambridge, UK). In brief, 96-well microtitre plates were coated with purified anti-BGC9331 polyclonal rabbit antibody (ICR, batch 8768, 8769). Following the addition of standards and patient samples, a conjugate of BGC9331 and horseradish peroxidase was added. Following further incubation and washing steps, the optical absorbance was read and plotted using a four-parameter logistic curve fitted by nonlinear regression as previously described (Aherne *et al*, 2001).

### Measurement of carboplatin by flameless atomic absorption spectrophotometry (FAAS)

Carboplatin was measured by FAAS. This method has been previously described (LeRoy *et al*, 1977). Samples were analysed on a Perkin-Elmer HGA800, AA300 Flameless atomic absorption spectrophotometer, and a Platinum Lumina HCC lamp (Perkin-Elmer, Beaconsfield, UK).

#### Preparation of calibration standards and quality controls (QCs)

Calibration standards were prepared individually for each assay batch by adding Carboplatin standards to control human plasma and ultrafiltrate. A blank was made using either plasma or ultrafiltrate only. A range of standards was made ranging from 5 to 50 ng ml^−1^ Pt (plasma samples) to 10–150 ng ml^−1^ Pt (ultrafiltrates).

For plasma analysis, three QCs were included in duplicate, ranging from low (15ng ml^−1^ Pt) to medium (25 ng ml^−1^ Pt) and high (45 ng ml^−1^ Pt), by diluting stock solutions with plasma. For ultrafiltrate analysis low (20 ng ml^−1^ Pt), medium (60 ng ml^−1^ Pt) and high (120 ng ml^−1^ Pt) QCs and stock solutions were diluted with plasma ultrafiltrate. A run was acceptable if four out of six QCs were within 15% of nominal concentration.

Control samples were used in each batch to check for interfering peaks of unknown origin and to measure 0 ng ml^−1^ Pt for the calibration curve. In the light of samples from the clinical study containing BGC9331, QCs spiked with BGC9331 were also included to be compared to QCs without and *vice versa*. PK analysis was performed using WinNonlin Software, version 3.2 (Pharsight Corp., CA, USA). The calculation of kinetic parameters was performed using a two- or three- compartment open model for BGC9331, and a noncompartmental model analysis for carboplatin. The following PK parameters were evaluated: maximum plasma concentration (*C*_max_), area under the plasma concentration–time curve (AUC), total body clearance (Cl), volume of distribution at steady state (*V*_ss_) and the terminal half-life (*t*_1/2_).

### PD assesment

#### High-performance liquid chromatography (HPLC) for dUrd

HPLC for dUrd and was performed using a previously defined method (Mitchell *et al*, 2000). This method has a limit of quantification of approximately 6 nM. In brief, the precipitating plasma proteins were removed by centrifugation and by the addition of perchloric acid, centrifugation and subsequent precipitation with 50 *μ*l 5 M KOH, and 250 *μ*l 3 M KHCO_3_. Samples were then recentrifuged and 200 *μ*l Na_2_HPO_4_ buffer was then added to the supernatant. This was followed by a solid phase extraction (SPE) step in which dUrd in the samples were eluted with methanol in SPE columns then evaporated to dryness in a vacuum, reconstituted in water and stored at −20°C pending analysis.

#### Estimation of dUrd recovery

Processing losses were estimated by adding 50 *μ*l 1/1000 diluted [5-^3^H]2′dUrd to triplicate 1.0 ml aliquots of normal human plasma. These samples were processed along with plasma samples. Stocks of labelled dUrd were assayed by HPLC every 3 months, and the value for dUrd activity was corrected for degradative losses. The activities of the processed standards was counted and compared to that of 50 *μ*l of the labelled dUrd to obtain a recovery factor, which was applied to all samples.

#### Data analysis

The peak area of dUrd was used as the assay parameter. A standard calibration curve was obtained from 1/*x* weighted least squares regression analysis, with the quality of fit evaluated by comparison of calculated to nominal values. Linearity of the calibration was confirmed using the correlation coefficient and comparison of the intercept with zero. Plasma dUrd results were expressed as the mean of the two duplicate assays after correction for recovery as above.

## RESULTS

### Patient characteristics

#### Demographics

Patient characteristics are shown in Table 2. In total, 14 patients were entered into the study between October 2000 and June 2002. The median age was 51 years (range 44–64). All patients had an ECOG PS of 0 or 1 on trial entry. Pathology was independently reviewed at the earliest possible stage of treatment at the RMH in all but one patient. Six patients had ovarian tumours of papillary histology, three mucinous, and of the remaining four patients, one had endometriod, one clear cell, one mixed mullerian and one undifferentiated tumour histology. A total of 66 cycles of chemotherapy were administered. The median number of cycles/patient was 6 (range 1–6). The mean number of cycles/patient was 4.7.

#### Previous chemotherapy

All patients had received 1–3 lines of previous chemotherapy and had a minimum platinum free interval of 6 months. Six patients received BCA second line. Four of these six received carboplatin/paclitaxel (CATAX), one received pegylated liposomal doxorubicin, carboplatin and paclitaxel (FATCAT), and one doxorubicin, carboplatin and paclitaxel (TCAT) as first treatment. Seven of the remaining eight patients received BCA third line, and the eighth patient received BCA fourth line. Prior chemotherapy regimens for these patients were carboplatin (CA), CATAX, epirubicin, cisplatin and 5-FU (ECF), the taxane analogue BMS184476, or single-agent liposomal doxorubicin.

### Dose escalations

Three patients were entered at dose level 1. No dose-limiting toxicities were seen at this level. A further three patients were entered at dose level 2. Five patients were entered at dose level 3 because the first two patients at this dose level received only day 1 of treatment due to non-treatment related complications. At dose level 4, two out of two patients experienced dose-limiting toxicities; therefore, dose level 3 was further expanded to a total of six patients and further PK and PD performed on patient 6 at dose level 3.

### Dose delays

One patient at dose level 1 (BGC9331 40 mg m^−2^) had a 1-week delay in chemotherapy due to asymptomatic neutropaenia. Similarly, two patients at dose level 2 (BGC9331 65 mg m^−2^) had a 1-week delay in either their 4th or 5th cycle of treatment. At the remaining dose levels, patients had either omission of their day 8 BGC9331 or a 1-week delay in one or two of the six cycles administered.

### Toxicities

The combination was generally well tolerated. There were no treatment-related deaths. Lethargy and nausea were the principal non-haematological toxicities, half of the patients experienced grade 1 or 2 lethargy and nine out of 14 patients experienced grade 1 or 2 nausea. Grade 1 or 2 alopecia occurred in 50% of patients. Diarrhoea affected two out of 14 and mucositis two out of 14 patients, but was short lived and did not require any treatment (Table 3). Grade 3 and 4 non-haematological toxicities were grade 3 lethargy in two patients at dose level 3 and one patient experienced grade 3 nausea at dose level 3. At dose level 4, one patient experienced grade 3 diarrhoea and one patient grade 4 nausea. At this dose level, one patient experienced grade 4 lethargy >7 days, which was a dose-limiting toxicity. Grade 1 or 2 neutropaenia and anaemia affected six out of 14 patients, respectively, with grade 1 and 2 anaemia affecting four out of 14 patients. No patients required platelet support or blood transfusion while on treatment.

No haematological or non-haematological grade 3/4 toxicities were seen at dose level 1. One patient had grade 3 neutropaenia at dose level 2. One patient had grade 3 neutropaenia at level 3. Dose-limiting grade 4 neutropaenia was seen in one patient at dose level 4. This patient was not admitted to hospital as there were no obvious symptoms or signs of infection, but was monitored daily by analysis of blood parameters. The toxicity was dose limiting by the fact that the neutropaenia was grade 4 for >10 days (Table 3).

### BGC9331 PKs

**Figure 2:**
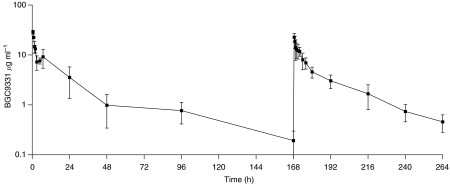
BGC9331 plasma concentration (mean±s.d. *μ*g ml^−1^) in three patients at dose level 2 (BGC9331 65 mg m^−2^).

No matrix effects were observed at these dilutions of drug in human plasma. Recovery of BGC9331 fortified into normal plasma (concentrations) was 121%. Interassay variation as determined using a control sample included in each assay was 20.1% (coefficient of variation). Recovery of BGC9331 from plasma was not affected by the presence of carboplatin (data not shown). PK measurements were performed at dose levels 1 and 2 (Figure 2). Mean clearance and *C*_max_ are shown in Table 4, and individual patient PK parameters for dose level 2 are shown in Table 5. Plasma BGC9331 levels were measured on a total of six patients at dose level 1 and 2 during their first cycle of chemotherapy. There was wide interpatient variability. The mean±s.d. total plasma clearance of BGC9331 after day 1 administration at 40 mg m^−2^ was 478 ml h^−1^±61.3. Following the day 8 dose the mean clearance±s.d. was 971 ml h^−1^±265. At dose level 2 of 65 mg m^−2^ BGC9331, the mean±s.d. total plasma clearance of BGC9331 after day 1 administration was 1197 ml h^−1^±580. Following the day 8 dose the mean clearance±s.d. was 746 ml h^−1^±584.

The mean±s.d. *C*_max_ of BGC9331 after day 1 administration at 40 mg m^−2^ was 11.21 *μ*g ml^−1^±2.51. Following the day 8 dose the mean *C*_max_±s.d. was 7.26 *μ*g ml^−1^±5.0. At dose level 2 of 65 mg m^−2^ BGC9331, the mean±s.d. value after day 1 administration was 16.54 *μ*g ml^−1^±2.4. Following the day 8 dose the mean *C*_max_±s.d. was 10.36 *μ*g ml^−1^±4.0.

### Carboplatin PKs

Data were unavailable for patient 2. The mean carboplatin AUC recovered for all patients was 5.1±1.1. Individual AUC measured for each patient are shown in Table 6.

### dUrd elevation as a PD end point of TS inhibition

**Figure 3:**
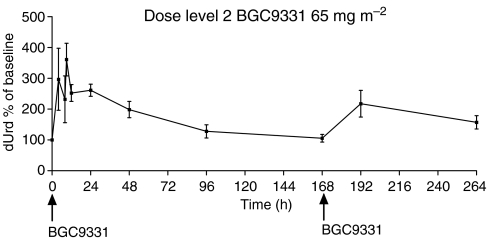
Mean dUrd for patients at dose level 2.

Results for one patient at dose level 3 of BGC9331 85 mg m^−2^ have not been included due to an error in sample preparation. There was a very wide interpatient variability in dUrd elevation in all patients studied (Figure 3). The mean pretreatment dUrd for all patients was 53±17 nM. This is consistent with previously published results (Plummer *et al*, 2003). The mean was similar for the three patients at dose level 1 (55±14 nM) and dose level 2 (49±25 nM). A rise of dUrd to approximately 300% of baseline was observed at dose level 1 (40 mg m^−2^ BGC9331) at approximately 12 h (mean elevation 312% of baseline) and persisted until 48 h (180±17 nM, 347±136%), at the next time point (96 h) the level was lower but still above baseline (198%). At dose level 2 (65 mg m^−2^ BGC9331) a rise of 360% above baseline was observed at 9 h, falling to 260% at 24 h and then 200% of baseline at 48 h and 128% at 96 h. The observed rise in dUrd above baseline 24 h following the day 8 BGC9331 dose was 225% at dose level 1 and 217% at dose level 2.

Deoxyuridine remained elevated above baseline up to 12 days after commencement of chemotherapy. The pattern of dUrd elevation observed is similar to that observed in previous published data (Plummer *et al*, 2003), although the patient numbers are small in this study.

### Antitumour activity and biological response

In total, 14 patients had measurable metastatic disease. Four out of 14 (28%) of patients had radiological evidence of antitumour activity. One patient had a complete response (CR) after four cycles of BCA. This patient had one line of previous chemotherapy and maintained a clinical and radiological remission for 4 years. Five out of 14 (36%) patients had a >50% reduction in their pretreatment CA-125 upon completion of treatment.

## CONCLUSION

BGC9331 is a rationally designed nonpolyglutamatable specific inhibitor of TS. It has demonstrated a wide range of antitumour activity *in vitro* and *in vivo* (Boyle, 1999). Phase I and interim analysis of phase II studies showed encouraging activity, particularly in heavily pretreated ovarian cancer (Plummer *et al*, 2003; Rader *et al*, 2003). This study was based on a number of rationales including preclinical evidence that impaired polyglutamation is a potential mechanism of resistance to antifolates (Jackman *et al*, 1995), and data from *in vitro* studies suggesting a reduced expression of FPGS in a panel of ovarian compared with colorectal cell lines (Jackman *et al*, 2002). BGC9331 does not require polyglutamation and therefore it was hypothesised that it may overcome this mechanism of resistance. A requirement of polyglutamation may possibly explain the poor clinical activity of the polyglutamatable TS inhibitor RTX in ovarian cancer (Gore *et al*, 1995; Muggia *et al*, 1998). In addition, for many solid tumours combining agents with different modes of action and different toxicity profiles offers the best chance of improving efficacy.

BGC9331 has been successfully combined with a number of agents in a phase I setting (Louvet *et al*, 2002; Wood *et al*, 2002; Benson *et al*, 2003; Bilenker *et al*, 2004). In our study it was found that patients only experienced dose-limiting toxicities approaching the full single-agent dose of BGC9331. This is supported by the relatively improved side effect profile of this combination as compared with other BGC9331 combination studies. For instance, when combined with topotecan at half the single-agent dose of both drugs, a high degree of haematological and non-hematological toxicity was observed, including a treatment related death (Benson *et al*, 2003). In combination with cisplatin the MTD was reached at 130 mg m^−2^ BGC9331 days 1 and 8, and cisplatin 75 mg m^−2^ on day 1 of a 21-day cycle. The predominant toxicity was grade 3 or 4 neutropaenia in three of the four patients at this dose level (Bilenker *et al*, 2004). The toxicity profile seen in our study was comparable to that reported in the BGC9331 monotherapy studies. In particular, in the phase II monotherapy study of BGC9331 130 mg m^−2^ given day 1 and day 8 in ovarian cancer, the main grade 3 and 4 haematological toxicities were neutropaenia and thrombocytopaenia, and the main non-haematological toxicities were nausea and asthenia (Rader *et al*, 2003). Thrombocytopaenia and neutropaenia were the main haematological toxicities in our study, with nausea and fatigue being the most common nonhaematological toxicities.

Dose-limiting toxicities were seen in two patients at dose level 4 (carboplatin AUC 5, BGC9331 100 mg m^−2^). These were grade 4 fatigue (patient bed bound for >50% of the day) for >7 days in one patient and grade 4 neutropaenia for >10 days. Myelosupression and fatigue are attributable to both drugs. Similar severe toxicities were seen in other combination studies with BGC9331, predominantly myelosupression and asthenia (Louvet *et al*, 2002; Wood *et al*, 2002).

Diarrhoea has been a feature of previous studies affecting 36% of patients as (Rader *et al*, 2003). Diarrhoea-related morbidity has also been a concern with RTX, but only one patient experienced grade 3 diarrhoea in our study at dose level 4 (BGC9331 100 mg m^−2^, carboplatin AUC5), which was self-limiting and the patient did not require hospital admission.

PK data for BGC9331 in our study is comparable to previous reports where the equivalent dosing schedule was administered (Plummer *et al*, 2003).The AUC and *C*_max_ for BGC9331 (Table 7) in our study are comparable with this historical published data at equivalent doses and schedules. However, some differences were observed such as the lower AUC of BGC9331 of 140 *μ*g h ml^−1^ in our study at 65 mg m^−2^ compared with 322 *μ*g h ml^−1^ at 69 mg m^−2^ (Plummer *et al*, 2003). However, the *C*_max_ at both comparable dose levels are similar in both studies, and the differences in AUC seen may be due to several reasons. Different methodologies were used in both studies (mass spectroscopy *vs* ELISA). The numbers of patients and the patient population in each study differed as the Plummer study had greater numbers and a more heavily treated traditional phase I population. The crossreactivity of the antibody did not lead us to believe that ELISA results should yield higher values than mass spectroscopy.

In this study, the measured carboplatin AUC is comparable to the target AUC5, and therefore there appears to be no PK interaction with BGC9331 at the doses used.

In a study comparing patterns of dUrd elevation between BGC9331 and other TS inhibitors (bolus/infusional 5-FU and RTX), different patterns of dUrd elevation are seen. With bolus and infusional 5-FU, smaller peaks are seen (235±125% bolus, 228±86% infusional) with the latter maintained throughout the infusional schedule. With the pure TS inhibitor RTX given on a 21-day cycle, a greater peak percentage rise is seen compared with 5-FU (349±128%) and reapproaches baseline at approximately day 15 (Ford *et al*, 2002).

We have shown PD evidence of TS inhibition by the combination of BCA. In a previous study of single agent BGC9331 at a similar dose level (42 mg m^−2^ days 1 and 8), the pattern of dUrd elevation was similar to our study, with dUrd rises seen after day 1 and day 8 in both studies (Plummer *et al*, 2003). The peak percentage rise in our study (BGC9331 at 40 mg m^−2^) after day 1 was 360% (9 h) compared with a peak percentage rise of 270% (72 h) (Plummer *et al*, 2003).

Despite wide interpatient variability in published studies, we have demonstrated similar rises in dUrd, indicating similar TS inhibition is achieved with the combination as with other TS inhibitors. However, as blood sampling times and patient numbers were not identical between studies, comparisons of dUrd elevation should be made with caution.

In the present study, there was radiological evidence of antitumour activity in 28% of patients, and 36% of patients had evidence of a response, as determined by falling CA-125 levels on treatment. However, it must be noted that the clinical activity observed in this setting may be attributable entirely to carboplatin. The response rate in this setting for single-agent platinum treatment ranges from approximately 19 to 30% (Gore *et al*, 1990; Markman *et al*, 1991). While dUrd elevation is achieved in our study, the clinical contribution of a TS inhibitor in combination would need to be determined in a future study by randomizing patients to carboplatin alone *vs* BCA. Incorporating assessment of relevant potential predictors of response (e.g. TS gene or protein expression) may also help to determine this contribution.

In conclusion, the combination of BGC9331 and carboplatin is well tolerated, with no significant PK interaction between the two drugs. There is evidence of TS inhibition with the combination and this is consistent with previous studies. We have demonstrated antitumour activity in platinum-sensitive relapsed ovarian cancer. Further exploration of this combination in this disease is warranted.

## Figures and Tables

**Table 1:** Dose levels of BGC9331 and carboplatin

**Dose level**	**BGC9331 (mg m^−2^)**	**Carboplatin**	**Number of patients**
−1	30	AUC 5	0
1	40	AUC 5	3
2	65	AUC 5	3
3	85	AUC 5	6
4	100	AUC 5	2
5	130	AUC 5	0

**Table 2:** Patient characteristics

Media age (range), years	51 (44–64)	
		
Histology	Papillary	6
	Mucinous	3
	Endometrioid	1
	Clear cell	1
	Mixed mullerian	1
	Undifferentiated	1
	Unknown	1
		
Prior lines of chemotherapy	One	6
	Two	7
	Three	1

**Table 3:** NCI-CTC grade 1–4 nonhaematological and haematological toxicities by dose levels (all cycles)

	**Dose level**			
	**1**	**2**	**3**	**4**
*Grade 1 and 2 toxicities*				
Lethargy	3/3	1/3	2/6	2/2
Vomiting	1/3	1/3	0/6	0/2
Nausea	2/3	3/3	2/6	2/2
Diarrhoea	0/3	0/3	2/6	0/2
Alopecia	1/3	2/3	2/6	1/2
Rash	0/3	0/3	2/6	1/2
Peripheral neuropathy	1/3	0/3	2/6	0/2
Mucositis	0/3	1/3	0/6	1/2
Anaemia	2/3	2/3	2/6	0/2
Thrombocytopaenia	0/3	1/3	1/6	2/2
Neutropaenia	1/3	1/3	2/6	2/2
				
*Grade 3 and 4 toxicities*				
Lethargy	0/3	0/3	2/6	1/2
Nausea	0/3	0/3	1/6	1/2
Diarrhoea	0/3	0/3	0/6	1/2
Thrombocytopaenia	0/3	0/3	0/6	0/2
Neutropaenia	0/3	1/3	1/6	1/2

**Table 4:** Mean plasma clearance and *C*_max_ BGC9331

	**Dose level 1**	**Dose level 1**	**Dose level 2**	**Dose level 2**
	**Day 1**	**Day 8**	**Day 1**	**Day 8**
Median plasma clearance (ml h^−1^)	478±61	971±265	1197±580	746±584
Median *C*_max_ (*μ*g ml^−1^)	11.2±2.5	7.3±5.0	16.5±2.4	10.4±4.0

Median plasma clearance in ml h^−1^±s.d. following day 1 and day 8 BGC9331 dosing at the first two dose levels in the study. The day 8 clearance at dose level 1 appears higher than that might be expected, and is not demonstrated at dose level 2. The *C*_max_ at dose level 2 is higher than that observed at dose level 1.

**Table 5:** Individual BGC9331 pharmacokinetics patients 4 to 6 at dose level 2 (BGC9331 at 65 mg m^−2^, carboplatin AUC 5)

**Patient**	**Administration**	**Dose level**	***T*_max_ (h)**	***C*_max_ (*μ*g ml^−1^)**	**AUClast (*μ*g ml h^−1^)**	**Vz_obs (ml)**	**HL_Lambda_z (h)**	**Cl_obs (ml h^−1^)**
4	1	2	0.5	16.8	90.4	31423.8	18.2	1197.5
	2		168.5	10.4	68.4	66088.6	27.8	1646.5
								
5	1	2	0.5	16.6	264.6	13891.3	25.5	378.4
	2		168.5	16.7	193.3	17208.1	21.7	549.2
								
6	1	2	0.5	12.6	66.5	101091.3	46.7	1499.8
	2		168.5	9.3	141.3	30257.3	28.1	746.0

**Table 6:** Free carboplatin pharmacokinetics

**Patient number**	**AUC (h *μ*g l^−1^)**	***C*_max_ (*μ*g l^−1^)**	**Alpha_HL (h)**	**Beta_HL (h)**	**CL (l h^−1^)**	**Vss (l)**	**V2 (l)**	**AUC in carbo**
1	29488.8	6815.3	2.2	50.3	8.7	123.3	91.2	**3.4**
3	55048.1	15973.2	1.2	8.2	4.8	27.1	15.1	**6.3**
4	46033.7	9539.7	2.4	100.9	7.0	186.5	158.5	**5.3**
5	54008.5	17397.3	1.5	92.0	6.0	115.6	101.0	**6.2**
6	42771.6	11427.8	1.8	60.4	6.7	109.7	88.9	**4.9**
14	38602.7	16744.5	0.4	2.6	6.1	16.5	8.6	**4.5**

Free carboplatin pharmacokinetics for two patients at dose level 1, three at dose level 2 and one patient at dose level 3. The figures in bold represent the measured AUC of carboplatin in the samples.

**Table 7:** Comparison of BGC9331 pharmacokinetics in BGC9331/carboplatin study with historical data from PI BGC9331 monotherapy study at comparable doses

**BGC9331 PI monotherapy study**			**BGC9331 and carboplatin PI study**		
**Dose BGC9331 (mg m^−2^)**	**AUC (*μ*g h ml^−1^)**	***C*_max_ (*μ*g ml^−1^)**	**Dose BGC9331 (mg m^−2^)**	**AUC (*μ*g h ml^−1^)**	***C*_max_** **(** *μ* **g ml^−1^)**
42	176	10.4	40	110	11.21
69	322	19.1	65	140	16.54
